# Choline—An Underappreciated Component of a Mother-to-Be’s Diet

**DOI:** 10.3390/nu16111767

**Published:** 2024-06-05

**Authors:** Agnieszka Dymek, Łukasz Oleksy, Artur Stolarczyk, Anna Bartosiewicz

**Affiliations:** 1 Students Scientific Club of Dietetics, Institute of Health Sciences, Medical College of Rzeszow University, 35-959 Rzeszow, Poland; 2 Department of Physiotherapy, Faculty of Health Sciences, Jagiellonian University Medical College, 31-008 Kraków, Poland; 3 Department of Orthopedics and Rehabilitation, Medical University of Warsaw, 02-091 Warsaw, Poland; 4 Institute of Health Sciences, Medical College of Rzeszow University, 35-959 Rzeszow, Poland

**Keywords:** choline, pregnancy, nutrition, infant development, dietary recommendations, women’s health, maternal diet, fetal growth

## Abstract

The nutritional status of the mother-to-be has a key impact on the proper development of the fetus. Although all nutrients are important for the developing baby, recent research indicates the importance of adequate choline intake during the periconceptional period, pregnancy, and lactation. Choline plays a key role in the biosynthesis of cell membranes, supporting liver function, neurotransmission, brain development, and DNA and histone methylation. Choline participates in the formation of a child’s nervous system, supports its cognitive development, and reduces the risk of neural tube defects. The human body is incapable of producing sufficient choline to meet its needs; therefore, it must be obtained from the diet. Current data indicate that most women in their reproductive years do not achieve the recommended daily intake of choline. The presented narrative review indicates the importance of educating mothers-to-be and thereby increasing their awareness of the effects of choline on maternal and child health, which can lead to a more aware and healthy pregnancy and proper child development.

## 1. Introduction

The diet of a mother-to-be is a key element in the proper development of the child. Although all macronutrients and micronutrients are essential for fetal growth, the American Academy of Pediatrics (AAP) identified choline as one of the key nutrients that supports the formation of the baby’s nervous system [1]. Choline is a water-soluble amine involved in a number of physiological functions that are important in all stages of human life. It acts as a methyl donor in the DNA and histone methylation processes, is a precursor of acetylcholine, a neurotransmitter that influences the proper functioning of the nervous system. It is essential for phospholipid synthesis, which is responsible, among other things, for the transport of lipids and the construction of cell membranes [2]. An adequate supply of choline during pregnancy reduces the risk of neural tube defects (NTD) and promotes child cognitive development and proper liver function [3]. Choline requirements increase markedly during gestation and lactation. Although it is available in a wide range of foods, current data indicate that most pregnant women do not reach the recommended daily intake of choline. Furthermore, commercially available prenatal supplements are often not enriched with choline or have a relatively low dose of choline [2,4].

In light of the growing scientific data that confirm the association of choline with fetal development, many researchers emphasize the need to update the official recommendations of obstetric societies, taking into account the need to supplement this component during the periconceptional period and during pregnancy and lactation. Furthermore, several researchers suggest that a higher intake (exceeding current recommendations) of this ingredient should also be considered to achieve optimal health benefits for the child and that education campaigns among women should be implemented immediately [2,5,6,7,8].

The purpose of this study is to review scientific publications on the importance of choline and the unique role of this component in the diet of mothers-to-be, which affects the normal course of pregnancy and the fetal development.

## 2. Materials and Methods

The article has a narrative nature. The scientific databases PubMed and Google Scholar were searched by entering the following keywords in various combinations: “choline”, “pregnancy”, “infant”, “fetus”, “one carbon metabolism”, “supplementation”, “early development”, “brain development”, “nervous system development”, “newborn health”, “maternal diet”, “gut microbiota”, “epigenetics”, “epigenetic mechanisms”, “DNA methylation”, “pregnancy complications”, “adverse pregnancy outcomes”.

The following nine topics were eventually identified: (1) definition of choline; (2) choline metabolism; (3) functions of choline; (4) effects of choline on fetal development; (5) effects of choline on epigenetic processes; (6) choline and the gut microbiota; (7) choline requirements; (8) choline supplementation; and (9) effects of choline on pregnancy complications.

The analysis mainly included meta-analysis, reviews, systematic reviews, observational studies, and clinical trials published in English. To provide the timeliness of the presented studies, the search results were limited to articles published between 2014 and 2024. However, in some cases, older studies have been cited. These studies add important aspects to the content of this work. Further relevant articles were identified by backward and forward searching.

**Figure 1:**
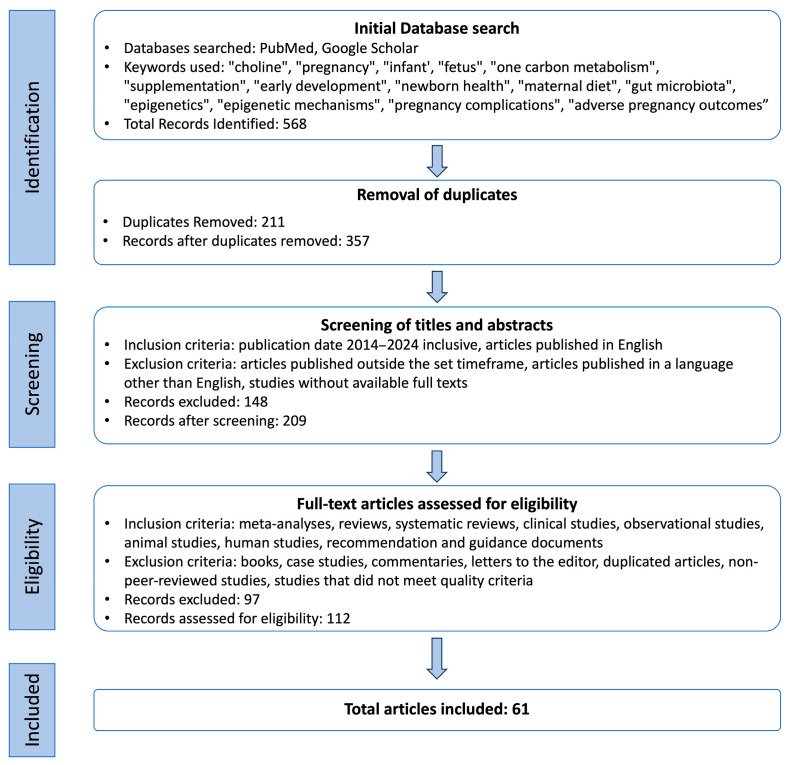
Flowchart for selection of methodology.

A total of 61 scientific sources were collected. Although this article is not exhaustive, it is a summary of the most important and recent information on the effects of maternal choline intake on child development and health. Figure 1 is an illustration of the search process for this narrative review.

## 3. Choline

Choline plays a key role in many metabolic and physiological processes, while also being an essential micronutrient for maintaining human health and development. Although choline can be derived by endogenous synthesis in hepatocytes, it is generally not high enough to cover the body’s needs. Therefore, its main source should be the daily diet [9]. A wide range of foods contain choline, but the highest levels are found in products of animal origin. Beef, chicken, pork, and fish provide more than 60 mg of choline per 100 g, while plant-based products, such as cruciferous vegetables, legumes, and nuts, provide at least 25 mg per 100 g [10]. Choline in food occurs in two forms: water-soluble (phosphocholine, glycerophosphocholine, and free choline) and fat-soluble (sphingomyelin, phosphatidylcholine (PC), and lysophosphatidylcholine). However, it usually takes the form of PC. Choline, in its water-soluble form (mainly present in breast milk), is transported into the portal circulation from where it reaches the liver. On the other hand, choline in its lipid-soluble form (present in most foods) is part of chylomicrons, which are then absorbed and distributed through the lymphatic system [11].

To date, choline requirements have not been precisely defined. However, choline’s key role in health was highlighted by the Food and Nutrition Board of the US National Academy of Sciences in 1998. The Adequate Intake (AI) values for choline was then set at 7.5 mg/kg bm/d and 425 mg/d for non-pregnant women. Higher AI was established for pregnant (450 mg/d) and lactating (550 mg/d) women to ensure that the placenta provides adequate choline to the developing fetus [12]. Due to insufficient data on the intake of choline from the population, the following standards could not be established: recommended daily allowance (RDA) and estimated average requirement (EAR) [2].

Current research suggests that choline is an undersupplied nutrient among European [13], Australian [14], and American [15] populations. Derbyshire et al. [2], in a review that considered data from studies conducted between 2004 and 2021, indicate that the average choline intake of women worldwide was lower than recommended by the guidelines. Furthermore, no higher choline intake was observed in studies of pregnant and lactating women compared to studies of non-pregnant women. These data suggest that the diets of the vast majority of women do not provide the recommended amount of choline.

## 4. Choline Metabolism

Among the metabolic transformations of choline, we can distinguish pathways involved mainly in the production of betaine, acetylcholine (ACh), trimethylamine (TMA), and phospholipids [9]. These processes occur mainly in the liver, except for the synthesis of the neurotransmitter ACh, which takes place in both cholinergic neurons and the placenta [4]. Vesicular ACh is then released into the synaptic gap, where it binds to receptors of the central and peripheral nervous systems [9].

**Figure 2:**
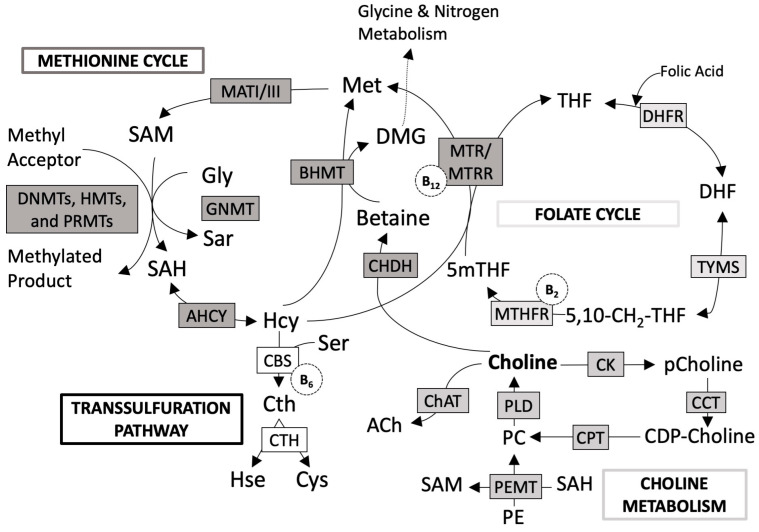
One carbon metabolism metabolic pathways [16]. Folate cycle metabolites and enzymes: 5,10-CH2-THF, 5,10-methylenetetrahydrofolate; 5mTHF, 5-methyl-tetrahydrofolate; DHF, dihydrofolate; DHFR, dihydrofolate reductase; MTHFR, 5,10-methylenetetrahydrofolate reductase; TYMS, thymidylate synthase. Methionine cycle metabolites and enzymes: DMG, dimethylglycine; Hcy, homocysteine; Met, methionine; SAH, S-adenosylhomocysteine; SAM, S-adenosylmethionine; Sar, sarcosine; Gly, glycine; AHCY, S-adenosyl-L-homocysteine hydrolase; BHMT, betaine-homocysteine S-methyltransferase; CHDH, choline dehydrogenase; GNMT, glycine *N-*methyltransferase; MATI/III, methionine adenosyltransferase; MTR, methionine synthase; MTRR, methionine synthase reductase; DNMTs, de novo and maintenance DNA methyltransferases; HMT, histone methyltransferase; PRMT, protein arginine methyltransferase. Transsulfuration pathway metabolites and enzymes: Cth, cystathionine; Cys, cysteine; Hse, homoserine; CBS, cystathionine β-synthase; CTH, cystathionine γ-lyase; Ser, serine. Choline metabolites and enzymes: CDP–choline, cytidine diphosphate-choline; PC, phosphatidylcholine; pCholine, phosphocholine; PE, phosphatidylethanolamine; ACh, acetylocholine; CCT, CTP:phosphocholine cytidylyltransferase; ChAT, choline acetyltransferase; CK, choline kinase; CPT, cholinephosphotransferase; PLD, phospholipase D; PEMT, phosphatidylethanolamine *N-*methyltransferase. Coenzymes: vitamin B2, B6 and B12.

In mitochondria, choline is oxidized to betaine, which, along with folate, is the donor methyl group in carbon one metabolism (1CM) (Figure 2) [16]. This pathway is particularly active under conditions of excess choline [4]. Homocysteine (Hcy) accepts a methyl group from betaine to form methionine. Then, methionine is converted to the universal methyl donor S-adenosylmethionine (SAM) and then to dimethylglycine (DMG) [17]. This is an alternative pathway to the vitamin B12-dependent homocysteine re-methylation pathway [9]. The methyl groups provided by SAM are used for histones and DNA methylation and to synthesize several metabolites (e.g., PC, hormones, neurotransmitters, creatine). DMG, together with sarcosine (which is a demethylated derivative of DMG), can provide monocarbon units in 1CM by folic acid [17]. When folic acid is deficient, liver choline stores are depleted, increasing the requirement for another methyl source, betaine [2].

Choline is also a precursor to phosphatidylcholine (PC). The production of PC takes place via the cytidine diphosphate (CDP)–choline pathway, which accounts for 70% of the total hepatic PC production. It can also be produced by de novo synthesis via methylation of phosphatidylethanolamine to PC. This reaction, catalyzed by phosphatidylethanolamine *N-*methyltransferase (PEMT), uses three SAM molecules, which produce three S-adenosylhomocysteine (SAH) molecules, homocysteine precursors. The activity of the hepatic PEMT pathway can result in the formation of up to 50% of the homocysteine produced. It is also the only known pathway by which endogenous de novo choline synthesis occurs in humans. Current data suggest that the PC molecule may differ depending on whether it has been generated by the PEMT or CDP–choline pathway [9]. PC derived from the CDP–choline pathway contains saturated fatty acids (C16, C18) and PC derived from PEMT contains long-chain polyunsaturated fatty acids (PUFAs) (C18–C22) [4]. Both types of molecules can be transported by VLDL from the liver into the circulation to reach various tissues, in particular the placenta. During the last trimester of pregnancy, there is increased activity of the CDP–choline and PEMT pathways. Interestingly, umbilical cord plasma is only enriched in PC products derived from the PEMT pathway, indicating a certain preference for transport of phosphatidylcholine to the developing baby. This could be explained by PC enrichment in DHA (C22:6 n3), a polyunsaturated fatty acid of the omega-3 family that accumulates in the neonatal brain during the third trimester of gestation. It should be noted that in studies of women of childbearing age, choline supplementation increased the activity of the PEMT pathway and led to a higher concentration of DHA in PC of erythrocytes. These results suggest that choline supplementation may have a beneficial effect in improving DHA bioavailability [4].

In the bloodstream, esterified forms of choline (sphingomyelin, phosphocholine, and PC) are lipoprotein components and free choline occurs in an unbound form. Unabsorbed choline can be converted to trimethylamine (TMA) by gut bacteria and then oxidized to trimethylamine *N-*oxide (TMAO) in the liver. Current data suggest that increased plasma TMAO levels may be associated with a higher risk of cardiovascular disease. However, more research is needed in this area [12].

## 5. Biological Functions of Choline

Choline has several key functions that support the development and health of the human body. Its derivatives, PC and sphingomyelin, are essential for the following processes: cell division and growth, lipid transport, cell membrane synthesis, myelination of nerve cell axons, and cell signaling [11]. As a precursor of ACh, choline has a direct effect on cholinergic neurotransmission [12]. Therefore, it plays a crucial role in brain development, influencing processes such as gliogenesis, neurogenesis, progenitor cell proliferation, and differentiation. As a donor of a methyl group during DNA and histone methylation, choline is believed to affect gene expression and, as a result, the coding of proteins involved in processes related to memory and learning. As with folic acid, it also plays a role in the conversion of homocysteine to methionine and helps regulate homocysteine levels in the body [10].

Choline is related to many metabolic pathways and inadequate dietary intake can have adverse health effects, including reduced cognitive function, muscle damage, homocysteinemia, non-alcoholic fatty liver disease (NAFLD), and carcinogenesis [12].

## 6. Effect of Choline on Fetal and Infant Development

Both the prenatal and early postnatal periods are considered “sensitive windows of development”. This is a time when the child’s body is particularly susceptible to certain stimuli from the environment that can permanently affect its development. In recent years, the important role of choline in the context of normal fetal development has been increasingly highlighted [2,8]. This component has several functions in the small body from the early stages of pregnancy, probably before the pregnant woman is aware of her condition. Therefore, it seems particularly important for women to ensure an adequate level of choline before and during pregnancy [3].

The results of a systematic review by Derbyshire et al. [5] suggest a positive impact of maternal and infant choline supplementation during the first 1000 days of life on child development. Benefits include support for nervous system development, improved cognitive function, and protection against abnormal metabolic and neural processes, especially when the fetus is exposed to alcohol. The demand for choline is notably elevated during both the fetal and neonatal period, and any damage to the small body resulting from choline deficiency can be permanent. Therefore, the authors point out that not providing choline within the first 1000 days of life can lead to lasting impairments in brain function.

Caudill et al. [18] conducted a randomized clinical trial (RCT) in which babies born to women supplemented with 930 mg of choline chloride per day during the third trimester of pregnancy, compared to babies of women who received the same supplement at 480 mg/d, showed higher levels of information processing, measured by visual attention task. Furthermore, after 7 years, the researchers made an additional observation and found that the children whose mothers supplemented with the higher dose of choline performed significantly better on a computerized color location memorization task. The results suggest a long-term beneficial effect of prenatal choline supplementation on the cognitive function of the offspring [19]. In 2022, the same researchers published a paper in which they evaluated the ability to focus attention on the 7-year-olds mentioned above [20]. To do this, they used a sustained attention task (SAT), consisting of 216 trials, during which the child had to respond correctly to a computer-generated signal. The results of this study showed that children of women who supplemented during pregnancy with a higher dose of choline (930 mg/d) were better at correctly detecting signals during the 12 min session than children born to women who received a dose of 480 mg choline/d. Children of mothers supplementing with choline at 930 mg/d scored higher on SAT, which may suggest that a high maternal choline intake has a beneficial effect on child concentration and attention. The small sample size is a major limitation of these studies; however, they provide convincing evidence of a persistent, significant effect of maternal choline intake on the cognitive development of the child.

On the contrary, an older RCT study by Cheatham et al. [21] does not support the above results. The researchers tested whether PC supplementation at 750 mg/d by women from the 18th week of pregnancy to the 90th day after delivery would increase the cognitive abilities of babies at 10 and 12 months of age. In both the control and study groups, there were no differences in spoken words, long-term episodic memory, short-term visuospatial memory, or child development scores using the Mullen Early Learning Scale. It is possible that the lack of cognitive benefit from increased maternal PC intake may have been due to inadequate adherence by study participants. A potential reason, the study authors point out, could also be that the infants were observed for too short a period (2 months) and that the supplement was administered in a form of PC, which has a different bioavailability compared to choline.

A recent systematic review and meta-analysis of human studies examined the relationship between choline intake by a pregnant woman and the risk of fetal neural tube defects (NTD) [7]. The results of this research suggest that insufficient choline consumption or low levels of this compound in maternal blood are associated with a 36% increased risk of the occurrence of NTD. The authors of the study add that the connection between inadequate choline consumption and the appearance of NTD is not influenced by folic acid levels, although it might be more pronounced in women experiencing vitamin B_9_ deficiencies. This may be due to the fact that both folic acid and choline contribute a methyl group during the conversion of homocysteine to methionine. When folic acid is abundant, choline can be conserved for other metabolic processes. Animal studies have indicated that supplementing the mother with choline prevents a greater number of cases of spina bifida than vitamin B_9_. Choline may have a distinct role in brain development and cannot be fully substituted for folic acid [7].

Choline, which acts as a precursor to phospholipids involved in removing triglycerides from the liver, appears to be essential for the proper functioning of this organ in the child’s body [8]. Studies in humans have shown that choline concentrations are high in breast milk and umbilical cord blood, confirming that fetuses and babies receive this component from their mothers [22,23]. In contrast, an animal study indicates that the elimination of choline from the diet of pregnant rats results in NAFLD in both the mother and the baby [24]. This is explained by the fact that the fetus and the newborn cannot synthesize choline in sufficient quantities to support regular liver function [8]. Although no human studies have yet been conducted on the effects of low maternal choline intake on liver function in the fetus or breastfed infants, the European Food Safety Authority (EFSA) recently approved a health claim on the significant role of choline in this aspect. As stated by the EFSA panel, scientific evidence has shown that “maternal choline intake during pregnancy and lactation contributes to normal liver function in fetuses and exclusively breastfed infants” [25].

## 7. Maternal Choline Intake and Fetal Epigenetic Regulation

Epigenetics is a relatively new field of science that deals with heritable changes in gene expression and function through modifications of the structure of the chromatin without altering the sequence of genetic codes. Epigenetic mechanisms can include DNA methylation (the addition of a methyl group to the fifth carbon of a cytosine), histone modifications (acetylation, methylation, ubiquitination, sumoylation and phosphorylation), modification of chromatin structure, and the involvement of microRNAs in altering gene expression [26]. Epigenetic programming begins in the early stages of fetal life, and the processes that determine it depend on environmental factors (including a diet rich in 1CM micronutrients) and are reversible [27,28]. Recent reports link epigenetic changes to normal function of the fetal nervous system [10,29,30]. The maternal supply of choline plays a key role in the epigenetic regulation that determines the normal development of the fetal and neonatal brain [31]. As mentioned above, choline is involved in 1CM as a methyl group donor. This allows the methionine cycle to produce betaine, then methionine and finally SAM, the main donor of methyl groups [16]. This is crucial to regulate gene expression because SAM provides methyl groups for enzymes such as DNA methyltransferase (DNMT) and histone methyltransferase (HMTS/KMT), which methylate DNA and histones, respectively [16,32,33]. DNA methylation is one of the most important epigenetic mechanisms in humans and affects the regulation of gene expression (activation/silence). It involves the transfer of a methyl group to the fifth carbon atom of the cytosine ring in DNA regions rich in CpG dinucleotides [34,35]. Deregulation of this mechanism can lead to abnormal attachment to the methyl group and consequently affect the function of the nervous system and related processes such as learning, memory, complex behavior, and others [35]. Histone methylation also regulates gene expression, but through changes in chromatin structure. This process occurs mainly at lysine (K) and arginine (R) residues on the *N*-terminal tails of histones and can alter the accessibility of stretches of DNA to the transcriptional machinery. Therefore, choline is involved in two important epigenetic reactions that affect gene expression: DNA methylation and histone methylation [30].

The fetal and neonatal periods are key periods for epigenetic programming, as the small organism is particularly sensitive and susceptible to any environmental changes [36]. In the context of normal nervous system development, a number of animal studies have been conducted to assess the importance of adequate maternal choline intake for the regulation of fetal epigenetics. Studies in rodents suggest that a lack of choline in the mother’s diet during a key period of fetal brain development can lead to a reduction in neuronal synthesis in areas of the hippocampus [37,38,39] and the cerebral cortex of the offspring [40,41]. In a study by Mehedint et al. [42] in pregnant mice, changes in histone methylation of fetal neuronal progenitor cells (NPCs) in the hippocampus were evaluated in relation to the amount of choline in the diet. In mice fed a diet deficient in choline between days 12 and 17 of gestation, it was observed that on day E17, the expression of the HMTase G9a gene, which is responsible for the methylation of lysine 9 in histone H3, was reduced in the ventricular and subventricular zones of the hippocampus. As a result, the amount of monomethylated lysine 9 in histone H3 (H3K9me1) was reduced by 25% and dimethylated lysine 9 (H3K9me2) by 37%. Therefore, histone H3 had fewer methyl groups near the repressor 1 (RE1)-binding site in the *Calb1* gene, which encodes the Calbindin 1, which plays an important role in regulating calcium levels in nerve cells. In addition, a CpG site in the promoter of this gene was found to be hypermethylated. This reduced the binding of transcription factor silencing repressor 1 (REST, RE1-Silencing Transcription Factor) to the RE1 sequence in the promoter of the *Calb1* gene. As a result, Calb1 gene expression increased in NPC under both in vivo and in vitro conditions. This study demonstrates that the availability of choline in the diet can affect neurogenesis in the hippocampus, leading to epigenetic changes in the fetal brain. A more recent study [40] investigated the effects of choline deficiency in the maternal diet on the development of the fetal structural cortex. For this purpose, the mothers of mice were fed two types of diets: a control (CT) and a low-choline diet (LC). Low levels of choline between the 11th and 17th day of mouse gestation were found to result in reduced expression of the epidermal growth factor receptor (EGFR), a protein involved in several aspects of the regulation of neural cell processes such as growth and differentiation. These changes led to a reduction in the number of two types of NPCs: radial glial cells and intermediate progenitor cells. In addition, the number of neurons in the upper layer of the cerebral cortex was lower in the offspring of mothers fed an LC diet both on day 17 of pregnancy and at 4 months of age. This work confirms the importance of inadequate maternal choline intake in the context of neurogenesis in the cerebral cortex of the offspring. Another study [43] investigated the effect of choline in the maternal diet on angiogenesis in the fetal mouse brain. In pregnant mouse fetuses fed a diet deficient in choline, a reduction in the number of blood vessels in the hippocampus was observed compared to offspring whose mothers’ diets were higher in choline. The authors of the study suggest that the diet deficient in choline of pregnant mice leads to reduced DNA methylation in the fetal brain in the region of two genes (*Vegfc* and *Angpt2*) that regulate the formation of blood vessels. Reduced methylation usually leads to increased activity of these genes, and as a result *Vegfc* and *Angpt2* are overactive in the fetal brain. This increases angiogenic signaling, which accelerates endothelial cell differentiation and inhibits cell division. The result is a reduction in the number of blood vessels in the hippocampus.

The results of animal models suggest a neuroprotective effect of maternal choline intake in the developing fetal brain. This component, through its participation in epigenetic mechanisms, influences the expression of genes involved in the normal functioning of the nervous system. At the same time, the above findings must be confirmed in human studies, as there are currently few data on the relationship between choline and epigenetic mechanisms in the human brain.

## 8. Maternal Choline and Gut Microbiota

The gut microbiota plays a key role in the health and function of the human body, reflecting the lifestyle and dietary habits of the host [44]. Gut bacteria have many functions in the human body, including digesting nutrients, supporting the immune and nervous systems, producing biologically active substances, and protecting against pathogens. Disorders in their composition, known as gut dysbiosis, have been linked to many diseases [45]. The gut microbiome is formed during fetal life [46] and its composition is influenced by many factors, including mode of delivery, medications, breast-feeding, formula feeding, maternal diet, maternal health, and perinatal weight. The mother’s gut microbiota therefore influences the growth and development of her baby [47].

The interaction between choline and the gut microbiota is an area of ongoing research. Gastrointestinal bacteria show the ability to metabolize this component via the choline utilization *(cut*) pathway, leading to the production of TMA and reducing the bioavailability of choline. Increased choline metabolism in the maternal bacterial population may limit fetal access to choline which, in combination with toxic TMAO, may affect fetal growth and lipogenic metabolism [48]. Inadequate levels of available choline, which is the precursor of SAM, can affect DNA methylation in the offspring. Furthermore, choline deficiency with increased TMAO can negatively affect epigenetic mechanisms in the mother’s body, especially during fetal development. This is because there is an increase in the production of reactive oxygen species (ROS), which can damage DNA. Such damage (base deamination/depurination) activates DNA repair mechanisms and contributes to the loss of cytosine methylation [48]. In addition, higher levels of TMAO are a risk factor for GDM in pregnant women [49]. However, not all bacteria can synthesize TMA from choline [50]. Bacteria in the genera *Actinobacteria*, *Proteobacteria,* and, in particular, *Firmicutes* have the genes needed to produce TMA [51]. As each individual has a different profile of gut bacteria, the level of TMAO synthesis can be highly individual. Furthermore, different forms of dietary choline have different bioavailability to microorganisms, so the contribution of these choline derivatives to the production of TMA is not identical [52]. For example, the intake of choline in the form of choline dihydrate was associated with a 3-fold higher plasma concentration of TMAO compared to PC [53]. These data suggest that the relationship between choline and the gut microbiota is extremely complex and highly individual.

The daily diet is known to have a major impact on the composition and function of the host’s gut microbiota. Recent scientific reports suggest that insufficient dietary choline can contribute to changes in gut microbiota composition and predispose to disease. This may be particularly relevant in the context of pregnancy status and fetal health. For example, in a study [54] of mice fed a diet deficient in choline, the development of NAFLD and gut dysbiosis was observed. After 2 weeks, there was a significant decrease in *Alistipes*, and after 4 weeks there was a decrease in *Bifidobacterium* and an increase in *Bacteroides*. On the other hand, a study in women [55] investigated the effect of dietary choline on the microbiota profile of the gut. It was found that dietary choline deficiency caused individual changes in the composition of the gut microbiota, including an increase in *Gammaproteobacteria* and *Erysipelotrichi*. These changes were associated with adverse changes in liver fatty tissue. Another recent study [56] shows that feeding pregnant rats a diet deficient in choline causes changes in the gut microbiota that are associated with phenotypes in both male and female offspring.

These data suggest that the relationship between choline and gut microbiota is extremely complex and highly individual. The choline content of the maternal diet appears to play an important role in shaping the composition and function of the gut microbiota, which may have health implications for both the mother and the child. Certainly, more research is needed in this area.

## 9. Maternal and Fetal Choline Requirements

As already mentioned, endogenous production of choline within the human organism occurs through the liver PEMT pathway and is insufficient to prevent deficiencies in this component. In premenopausal women, estrogen induces the expression of *PEMT*, the gene that encodes the enzyme that catalyzes de novo choline biosynthesis. During gestation, estrogen concentrations increase from about 1 to 60 nmol/L at term, indicating an increase in endogenous choline production during this time. Therefore, expectant mothers benefit from a natural defense against choline deficiency and are less dependent on dietary sources of choline. This mechanism ensures elevated concentrations of choline during pregnancy and lactation, periods when demand for this component is particularly high. However, the increased activity of *PEMT* in women may not fully compensate for the inadequate dietary intake of choline [6]. Previous observations have shown that choline crosses the placenta into the child’s body, and its serum or plasma levels are approximately 6–7 times higher in the fetus and the newborn compared to adults [5]. Transport of choline to the fetus from the mother’s body results in significant losses in the plasma of the pregnant woman; therefore, despite the increased ability to synthesize choline, the requirement is so high that the mother’s stores are used. This is supported by the work of Yan et al. [57], who investigated the effect of pregnancy and maternal choline intake on methyl donor concentration (choline derivatives). It was observed that regardless of the level of choline intake, pregnant women had significantly lower mean concentrations of: DMG (down 38%), sarcosine (down 49%), and betaine (down 55%) compared to nonpregnant women. Such a large decrease in the concentrations of these metabolites is due in part to the increased use of betaine as a methyl donor while at the same time reducing its production as a result of the preferential diversion of choline toward the CDP–choline pathway in PC synthesis. In fact, during pregnancy, one-carbon metabolism is increased to promote cell proliferation and support epigenetic processes. Reduced choline-derived methyl donor supply may interfere with homocysteine re-methylation (leading to hyperhomocysteinemia and SAM deficiency) and lead to secondary folate deficiency due to compensatory supply of methyl groups for 1CM. It should be noted that increased choline intake during pregnancy can improve the metabolic rates of this nutrient. In the same study [57], the consumption of 930 mg of choline per day by women in the third trimester of pregnancy, compared to 430 mg, resulted in higher levels of choline-derived methyl donors. This intake also helped to rebalance the competition between the betaine and CDP–choline pathways, both of which use choline as a substrate, making it more similar to the balance found in nonpregnant women [58]. These results indicate that a 480 mg choline intake may be insufficient to meet the demands of pregnancy and show that a higher maternal choline intake increases the use of choline as a methyl donor in the child and mother’s body. Since breast milk contains large amounts of choline, lactation further increases the mother’s need for choline, further depleting tissue stores.

Choline requirements depend on single nucleotide polymorphisms (SNP), which can influence the use of choline as a methyl donor, for the production of betaine or PC synthesis, as well as the allocation of choline between the PEMT and CDP–choline pathways [5].

As a result, researchers are concerned that choline AI for pregnant women of 450 mg/d is inadequate and can lead to negative health outcomes for both the child and the mother [5]. None of the RCTs conducted to date with healthy pregnant women have shown negative effects of choline supplementation within the range of 550–930 mg/d, but they have shown benefits. On the contrary, the tolerable upper intake level (UL) of choline for adults is 3500 mg/d. This value was established to prevent fishy body odor and hypotension. There may be concern that gut bacteria can convert choline into TMAO, a potential risk factor for cardiovascular disease. However, it is unknown whether TMAO plays a causal role in the development of chronic diseases in humans, and the long-term effects of choline intake on health and TMAO concentrations in mothers and their children require further study [4].

## 10. Choline Supplements

Although it is advisable in the first place to meet nutrient requirements through diet, in situations of increased need and difficulty in obtaining a particular nutrient through diet, it is worth considering supplementation. In the case of choline, there are several preparations containing different forms of choline: glycerophosphocholine (GPC), choline bitartrate, choline chloride, lecithin (PC), and citicoline (CDP–choline) [59]. However, studies suggest that each of these forms provides a different amount of choline cation [60] and may have different dietary effects due to differences in bioavailability and physiological effects [61].

GPC is a very popular dietary supplement due to its high choline content (1000 mg provides 400 mg of choline) and its ability to cross the blood-brain barrier. The compound is also a precursor of ACh and, together with phosphocholine, is one of the major sources of choline for the fetus in breast milk [62]. Numerous studies suggest that GPC may have neuroprotective effects [63,64,65].

Choline in the form of choline bitartrate and choline chloride is also commonly used as a supplement. These products contain 410 mg and 746 mg of choline per 1000 mg, respectively [60]. Recent studies in adults and rats found that choline supplements in the form of choline chloride salt or choline bicinate contributed to higher TMAO production compared to PC [53,66].

A phosphatidylcholine supplement provides about 130 mg of choline per 1000 mg [60]. This compound, unlike the forms of choline mentioned above, is a lipid compound and has a different metabolic conversion. PC can be absorbed by the human body in two ways: unchanged or after being broken down to GPC by pancreatic lipases [67]. In its unaltered form, PC is transported directly to the lymphatic system, bypassing the liver. Approximately 50% of PC is absorbed in this way. When PC is broken down by pancreatic enzymes, GPC is formed, which is water soluble and enters the portal vein and then the liver. There, it can be converted to betaine or reused for PC synthesis [61]. Therefore, choline in the PC form may have different biological effects than its free form. Furthermore, as mentioned above, PC does not contribute to the increase in TMAO in blood plasma, which is responsible for the fishy body odor [21].

A recent study [68] investigated how four different choline supplements (choline chloride, choline bitartrate, GPC, and PC from egg) affected plasma concentrations and kinetics of: choline, betaine, and TMAO. All formulations were observed to increase choline and betaine levels to a similar extent (in about 1–2 h), with a delayed increase in the concentration of these metabolites about 3 h after PC treatment. This delay is due to the uptake of choline from PC, which involves the degradation of PC in the duodenum to free fatty acids and lyso-PC. Furthermore, all water-soluble forms of choline, but not PC, increased plasma TMAO concentrations. The highest TMAO levels were observed for choline dihydrate. However, it should be noted that only men participated in the study because, as the authors of the article point out, in women, estrogen could affect the activity of PEMT and thus the metabolism of choline.

As different forms of choline have different bioavailability and metabolism, more studies in women are needed to compare the available forms of choline for their health effects.

## 11. Maternal Choline Deficiency and Adverse Pregnancy Outcomes

In addition to the wealth of scientific evidence demonstrating the role of high levels of choline in the maternal diet for fetal development, there have also been several recent publications that highlight the association of maternal choline intake with adverse pregnancy outcomes. A recent meta-analysis [69] suggests that an inadequate supply of this component in the diet of pregnant women may be a risk factor for preterm birth, small for gestational age (SGA), preeclampsia (PE) and gestational diabetes mellitus (GDM).

Previous studies have shown that genes may influence the risk of GDM. In particular, the *CDKAL1* gene appears to have a strong association with the mechanism of this disease, and the work of Wang et al. [70] provided evidence that a polymorphism of this gene, i.e., rs7747752, is associated with the appearance of GDM in pregnant Chinese women. In contrast, a more recent study by Wang et al. [71] involving 207 Chinese women examined the interaction between this polymorphism and low plasma levels of L-carnitine, choline, and betaine for GDM risk. In women with the *CDKAL1* rs7747752 CC/CG genotype, low serum levels of L-carnitine and choline were found to promote the appearance of GDM. McArthur et al. [72] investigated the association of TMAO and its precursors (including choline) with GDM and PE in 1496 women. Mothers with high plasma choline concentrations were shown to have a lower risk of these complications, but the results were not statistically significant. On the contrary, in a study [73] of women with twin pregnancies, they found no effect of maternal choline levels on the risk of GDM.

Excessive secretion of sFLT1 and endoglin plays an important role in the pathogenesis of PE. These are anti-angiogenic factors produced by the placenta during pregnancy and then transported into the maternal circulation. They combine with angiogenesis-stimulating factors such as vascular endothelial growth factor (VEGF) and placental growth factor (PGF) to inhibit maternal endothelial function. This can lead to protein in the urine, hypertension, and preeclampsia [74]. A randomized controlled trial by Jiang et al. [75], involving women in their third trimester of pregnancy, tested the effect of choline supplementation at 450 mg/d and 930 mg/d on sFLT1 production. In the group of women taking higher dose of choline, there was a 30% reduction in sFLT1 in placental tissue and maternal blood compared to the group taking 450 mg of choline. These results were also confirmed in vitro using human trophoblast cell cultures.

The relationship between maternal choline intake and SGA in the newborn has not yet been well established. In an observational study conducted by Hoffman et al. [76], the authors suggested that pregnant women with plasma choline levels less than 7 µM had an almost 17-fold increased risk of having a baby with SGA compared to women with higher plasma concentrations of this component. Further research is needed in this area.

Regarding the effect of maternal choline on the risk of preterm birth, a study [77] published in 2013 that analyzed maternal nutrition in relation to the risk of preterm birth found no association. On the contrary, a recent study [78] involving 145 Chinese women who had preterm births and 157 women who had normal births found different results. A significant interaction was observed between choline intake and the rs7946 polymorphism of the *PEMT* gene in relation to the risk of preterm birth. Women with the AA genotype of the rs7946 polymorphism who consumed less than 255 mg of choline/d during pregnancy had an almost 4-fold higher risk of preterm birth compared to women with the GG genotype who consumed more choline per day. In addition, women with the AA genotype who had low choline levels also had elevated homocysteine levels, which have been shown to be associated with preterm birth [69].

It should be noted that there is still a lack of research on choline in preventing adverse pregnancy outcomes. The available data are relatively recent and have limitations. However, choline seems to play an important role in the normal course of pregnancy.

## 12. Conclusions

The importance of sufficient choline intake during pregnancy and lactation, particularly in relation to the proper development of the child’s nervous system, is highlighted in research involving both humans and animals [18,19,20,21,22,23,24,57,58]. Although the endogenous synthesis of choline in the body of the pregnant woman increases markedly, the demand for fetal foods is so high that it leads to depletion of maternal stores, becoming an essential component of the daily diet. The results of research indicate that choline supplementation during these critical periods of child development has been shown to promote the formation of the nervous system, improve cognitive function, and affect the normal course of neural and metabolic processes [6]. Furthermore, to gain a comprehensive understanding of the impact of choline on maternal and child health, the review includes information on the role of choline in the context of epigenetic mechanisms, pregnancy complications, interactions with the gut microbiota, and different forms of choline in supplement form. However, it should be noted that the studies published to date have several limitations (including a small study sample, and a short follow-up time) and there is still a need for high-quality RCT studies in this aspect. However, researchers question the validity of current standards for choline, suggesting that an increased supply of choline may be necessary to promote optimal neurological development in the child. Korsmo et al. [4] indicate that a daily choline supply of 450–1000 mg appears to improve brain function and the development of the fetal nervous system. Taking into account the crucial role that choline plays, even in the early stages of pregnancy, it is important to ensure an adequate supply of choline even when planning conception [3]. However, concerning data indicate that a significant percentage of women do not meet AI choline requirements, and the global trend of limiting animal-derived foods while lacking conscious supplementation is only exacerbating the already pronounced deficiency. There is a great need to increase educational efforts among women in their childbearing years and healthcare professionals about the importance of choline for proper fetal development [2].
